# Single-molecule dissection of CFTR folding defects and pharmacological rescue

**DOI:** 10.1073/pnas.2528216123

**Published:** 2026-03-31

**Authors:** Sang Ah Kim, Jesper Levring, Jue Chen, Tae-Young Yoon

**Affiliations:** ^a^School of Biological Sciences and Institute for Molecular Biology and Genetics, Seoul National University, Seoul 08826, South Korea; ^b^Laboratory of Membrane Biology and Biophysics, The Rockefeller University, New York, NY 10065; ^c^HHMI, Chevy Chase, MD 20815

**Keywords:** ΔF508 CFTR, membrane protein folding, single-molecule magnetic tweezers, pharmacological rescue

## Abstract

Cystic fibrosis is a lethal genetic disorder caused by misfolding of the cystic fibrosis transmembrane conductance regulator (CFTR) protein, most commonly due to the ΔF508 mutation. Despite extensive study, CFTR’s folding process has remained inaccessible to direct observation. Here, we apply single-molecule magnetic tweezers to resolve the complete folding trajectories of wild-type and ΔF508 CFTR with near–amino acid resolution. We find that CFTR follows a hierarchical, template-guided folding pathway in which N-terminal domains scaffold downstream folding. This mechanism tightly couples the free energy states of intermediates, allowing ΔF508-induced instability to propagate across the folding pathway. Pharmacological correctors, in synergy with ATP, reshape the entire folding energy landscape by catalyzing transitions rather than simply stabilizing end states. These long-range, allosteric effects reveal a folding-embedded regulatory network. Our work provides a quantitative framework for mapping multidomain protein folding and therapeutic rescue, offering a broadly applicable strategy for interrogating rare mutations and accelerating structure-based drug discovery.

## Significance

Misfolding of cystic fibrosis transmembrane conductance regulator (CFTR) underlies cystic fibrosis, and its complex, multidomain architecture makes it an ideal model for understanding how membrane proteins fold and how small molecules can restore native structure. Using single-molecule magnetic tweezers, we reveal how local instabilities propagate through CFTR’s folding pathway and show that pharmacological correctors act by catalyzing specific folding transitions in addition to stabilizing the native fold. These insights establish CFTR as a paradigm for dissecting folding mechanisms in large membrane proteins and for developing general strategies to correct misfolding across diverse human diseases.

The cystic fibrosis transmembrane conductance regulator (CFTR) is a vital member of the ATP-binding cassette transporter family uniquely functioning as an anion channel, conducting chloride and bicarbonate across epithelial cell membranes (1, 2). CFTR plays a key role in maintaining the hydration and pH of epithelial secretions, which is critical for physiological function and tissue homeostasis (3–5). Structurally, CFTR comprises an N-terminal lasso motif, two transmembrane domains (TMDs) that form the anion conduction pore, two cytoplasmic nucleotide-binding domains (NBDs) responsible for ATP binding and hydrolysis (6), and a regulatory (R) domain whose phosphorylation is required for channel activation (7).

Mutations in the *cftr* gene lead to cystic fibrosis, a severe autosomal recessive disorder characterized by salty sweat, chronic respiratory infections, and progressive pulmonary damage (8–10). CFTR mutations are categorized into six classes based on their impact on protein synthesis, folding, gating, and stability (11, 12). The most prevalent disease-causing variant is deletion of phenylalanine at position 508 (ΔF508), which is present in at least one allele in approximately 90% of individuals with cystic fibrosis (13, 14). This mutation disrupts proper protein folding, impairs trafficking to the cell surface, and reduces protein stability, ultimately leading to premature degradation by cellular quality control mechanisms (14–17).

The advent of CFTR modulator therapies has transformed cystic fibrosis treatment by targeting the molecular defects in mutant CFTR proteins. These small-molecule therapies include potentiators, which increase the channel open probability, and correctors, which enhance folding efficiency and promote trafficking of mutant CFTR to the plasma membrane (18–22). Dual-combination regimens of ivacaftor with lumacaftor or tezacaftor, and more recently the triple combination including elexacaftor, have markedly improved clinical outcomes (23). While these therapies now provide substantial clinical benefit to patients with cystic fibrosis, our understanding of the precise mechanisms by which these modulators act, and the extent to which they effectively rescue CFTR folding and function, remains limited.

CFTR biogenesis has been extensively studied using a variety of methods, including pulse–chase (en rule), proteolytic susceptibility assays, antibody mapping, and hydrogen–deuterium exchange mass spectrometry (24–28). In parallel, structural studies using cryogenic electron microscopy (cryo-EM) have resolved WT CFTR and the ΔF508 variant in distinct conformational states and identified the binding sites of pharmacological molecules (29–35). Despite these progresses, important questions remain regarding the precise kinetics of translation and folding, the thermodynamic landscape governing folding transitions, and how nascent CFTR is triaged between productive folding and degradation pathways in the context of disease-causing mutations or pharmacological intervention.

To bridge this crucial knowledge gap and gain a deeper understanding of CFTR folding dynamics, we employed single-molecule magnetic tweezers (MT). This biophysical technique offers unparalleled resolution in dissecting the folding landscapes of individual protein molecules real-time, an approach previously validated in studies of other complex membrane proteins (36, 37). By precisely modulating the mechanical force applied to tethered CFTR molecules, we reconstructed the folding and unfolding pathways of both WT and ΔF508 CFTR with near-amino acid resolution, capturing the kinetics and thermodynamics of transient intermediate states that are otherwise inaccessible. This analysis revealed a hierarchical, template-guided folding mechanism in which N-terminal domains fold early and serve as scaffolds to guide the proper folding of downstream regions. Notably, the destabilizing effects of the ΔF508 mutation—and the restorative actions of pharmacological correctors—were found to propagate and amplify along this folding pathway, such that the structural rigidity of early-folding domains directly influenced the folding efficiency of subsequent segments. The quantitative insights reveal that distinct combinations of correctors, in conjunction with ATP, actively catalyze folding transitions and function as allosteric modulators that reshape the CFTR folding energy landscape.

## Results

### Reconstruction of Full Unfolding and Refolding Processes of CFTR with Single-Molecule MT.

**Fig. 1.**
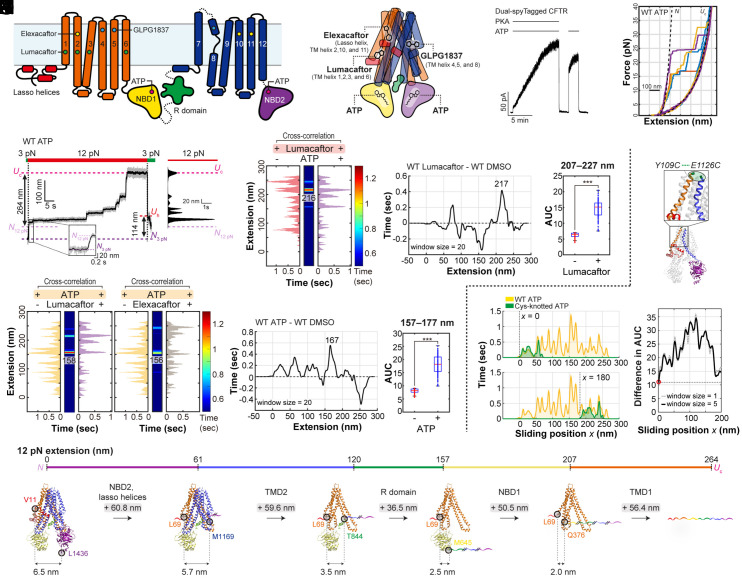
Single-molecule MT assay for CFTR unfolding. (*A*) Schematic of CFTR domain organization, highlighting the lasso helices (red), TMD1 (orange), NBD1 (yellow), R domain (green), TMD2 (blue), and NBD2 (purple). Individual TM helices are numbered, and the approximate membrane boundaries are indicated. Colored dots indicate the approximate locations of ligand-interacting regions: elexacaftor (yellow), lumacaftor (green), GLPG1837 (blue), and ATP (red) (29). (*B*) Structural representation of CFTR highlighting the binding sites of small-molecule modulators within the folded tertiary structure of CFTR (29). (*C*) Representative recordings of protein kinase A (PKA)- and ATP-dependent currents from dual-SpyTagged CFTR in inside-out excised patches. (*D*) force-extension curve (FECs) showing unfolding of single CFTR proteins under mechanical tension. Colored FECs represent example traces of individual force-ramp cycles, whereas black dotted lines indicate theoretical FECs for *N* and *U*_c_ states. (*E*) Example force-jump cycle (*Left*) and corresponding histogram at 12 pN (*Right*). Raw data of the exemplary trace at 1.2 kHz are shown in gray and 10-Hz median filtered data in black. (*F*) Cross-correlation analysis of unfolding histograms with lumacaftor and with or without ATP. *n* = 64 (WT Lumacaftor) and *n* = 47 (WT ATP + Lumacaftor). (*G*) Sliding window difference analysis comparing unfolding histograms of WT CFTR in the presence of lumacaftor vs. DMSO. *n* = 64 (WT Lumacaftor) and *n* = 45 (WT DMSO). (*H*) Comparison of AUC in the 207 to 225 nm extension range with versus without lumacaftor. (*I*) Cross-correlation analyses of unfolding histograms with ATP and with or without folding corrector. *n* = 64 (WT ATP), *n* = 47 (WT ATP + Lumacaftor), and *n* = 54 (WT ATP + Elexacaftor). (*J*) Sliding window difference analysis comparing unfolding histograms of WT CFTR in the presence of ATP vs. DMSO. *n* = 54 (WT ATP) and *n* = 45 (WT DMSO). (*K*) Comparison of AUC in the 157 to 175 nm extension range with versus without ATP. (*L*) Molecular structure of CFTR illustrating the engineered disulfide bond between Y109C and E1126C. The inset depicts a schematic of the disulfide-linked residues. (*M*) Comparison of unfolding histograms for Cys-knotted CFTR (*n* = 45) and WT CFTR in the presence of ATP. 5-nm sliding window, one-fourth the width used for full-length WT histograms to accommodate the shorter unfolding range of the Cys-knotted, was applied construct and aligned at two alignment positions: *x* = 0 nm (*Top*) and *x* = 180 nm (*Bottom*). (*N*) Quantification of AUC differences between Cys-knotted and WT CFTR unfolding histograms. The Cys-knotted histogram, averaged with 5-nm (dashed line) and 1-nm (dotted line) sliding windows, was shifted in 1-nm increments across the WT ATP histogram to calculate AUC differences. (*O*) Proposed CFTR unfolding pathway. Numbers above unidirectional arrows indicate the extension changes associated with each domain’s unfolding transition. Numbers between bidirectional arrows represent the end-to-end distances of structured regions, with circles marking the N- and C-terminal residues involved in tertiary structure formation.

CFTR is a large membrane protein composed of 1,480 residues arranged into five domains with a complex domain-swapping topology (Fig. 1 *A* and *B*). We first conducted a series of pilot experiments to test if it is feasible to use single-molecule MT assays to study CFTR unfolding and refolding. To tether CFTR in the MT, we used SpyTag-SpyCatcher system, an engineered peptide-protein pair that forms a spontaneous and irreversible isopeptide bond (38). SpyCatcher was conjugated to the DNA handles and SpyTags were introduced at both termini of the full-length human CFTR (hereafter referred to as WT and ΔF508, denoting the WT and ΔF508 CFTR constructs tagged with SpyTags), enabling stable and site-specific covalent attachment of each handle to the N and C termini during force application (*SI Appendix*, Fig. S1*A*). Electrophysiological analysis of the dual-SpyTagged CFTR revealed phosphorylation- and ATP-dependent currents typical of CFTR (Fig. 1*C*) and sensitivity to CFTR modulators, including the potentiator GLPG1837 and the inhibitor CFTR_inh_-172 (*SI Appendix*, Fig. S1 *B* and *C*). Furthermore, the CFTR correctors, lumacaftor and elexacaftor, enhanced the expression of the dual-SpyTagged variant, showing an additive effect similar to that observed with WT CFTR (*SI Appendix*, Fig. S1*D*). These findings indicate that the dual-SpyTagged variant preserves the functional and folding properties of WT CFTR and is therefore suitable for MT studies.

To prepare CFTR for single-molecule studies, we purified the dual-SpyTagged protein and attached two 512-base-pair DNA handles via SpyTag-SpyCatcher linkages (*SI Appendix*, Fig. S1 *E*–*H*). One DNA handle was anchored to the surface of a polyethylene glycol (PEG)-passivated flow cell, whereas the other was linked to a 3 μm-diameter magnetic bead (*SI Appendix*, Fig. S2*A*) (39). Magnetic force was applied to individual CFTR molecules reconstituted in a lipid bicelle environment, cycling gradually between 1 pN and 40 pN (*SI Appendix*, Fig. S2 *A* and *B*). The distance between the two SpyTags, representing the N and C termini (hereafter referred to as the extension), was monitored as a function of force (*SI Appendix*, Fig. S2*B*). The experimentally obtained FEC was compared to theoretical FECs for the native (*N*) and fully unfolded polypeptide (*U*_c_) states of CFTR (Fig. 1*D* and *SI Appendix*, Fig. S2 *C* and *D*). After repetitive force apply, CFTR proteins extended to yield a FEC matching the predicted trajectory for the *U*_c_ state, lacking any secondary structure at forces exceeding 15 pN (*SI Appendix*, Fig. S2 *C* and *D*; see *Methods* for extension value calculations). When the force was subsequently reduced to 1 pN and held for a 6-min incubation, CFTR refolded to the *N* state, evident by the FEC of the subsequent cycle aligning with the theoretical curve of a completely folded CFTR (Fig. 1*D*).

Encouraged by these observations, we proceeded to establish complete unfolding and refolding pathways under conditions of constant force. In these experiments, we started with CFTR in the *N* state with an initial force of 3 pN, then rapidly increased the force to 12 pN to initiate unfolding (Fig. 1*E* and *SI Appendix*, Fig. S2*G*). With a constant force of 12 pN, the extension of CFTR increased in a stepwise manner, reaching a maximum extension increase of 264 nm, which closely matched the theoretical value for complete unfolding from the *N* to the *U*_c_ state (Fig. 1*E* and *SI Appendix*, Fig. S2 *E* and *G*). The intermediate extension values observed during this process likely represent progressive unfolding of distinct structural elements. After CFTR reached the *U*_c_ state, we reduced the force to 3 pN to allow refolding while preserving the high-resolution capabilities of single-molecule MT for tracking this process (*SI Appendix*, Fig. S2 *H* and *I*) (36, 37, 40). At 3 pN, the extension of CFTR gradually decreased, with a maximum contraction of 114 nm, precisely matching the expected value for the *N* state (*SI Appendix*, Fig. S2 *F*, *H* and *J*).

These data demonstrated that the complete unfolding and refolding processes of CFTR could be faithfully reconstituted using our single-molecule MT assay. It was however noteworthy that not all trials resulted in successful refolding. As we will discuss later, the probability of refolding varied between WT and mutant CFTR and was influenced by ligands such as ATP and folding correctors. To gauge the robustness of the assay, we performed blinded experiments in which each trace was acquired in the presence of lumacaftor, elexacaftor, the potentiator GLPG1837, or a DMSO control. DMSO and GLPG1837 produced virtually identical refolding probabilities (~10%), whereas lumacaftor and elexacaftor more than doubled this value (>20%) (*SI Appendix*, Fig. S3*A*). The clear separation between correctors and noncorrectors highlights the sensitivity of the assay.

### The CFTR Unfolding Pathway.

Unfolding intermediates of WT CFTR were identified by comparing 12-pN unfolding histograms recorded with or without various ligands, with the assumption that each ligand primarily stabilizes the structural region to which it binds. Although each high-force pulling stochastically samples a different set of intermediates at 12 pN, the cumulative histogram converged after inclusion of more than 30 traces (*SI Appendix*, Fig. S3*B*). Indeed, 100 independent resampling of 35 traces drawn from the full dataset (~50 traces) produced virtually identical histograms with negligible variance (*SI Appendix*, Fig. S3 *B*–*E*). Through pairwise cross-correlation analyses, we identified intermediates that were elevated in both histograms due to a common ligand (Fig. 1 *F* and *I* and *SI Appendix*, Fig. S3 *F*–*H*; see *Methods* for the cross-correlation analysis). For example, the highest cross-correlation peak between two histograms obtained in the presence of lumacaftor occurred at 216 nm (Fig. 1*F* and *SI Appendix*, Fig. S4*A*), suggesting that lumacaftor stabilizes an intermediate with an extension of approximately 216 nm. Theoretical calculations indicated that the fully extended CFTR at 12 pN reaches 264 nm (*SI Appendix*, Fig. S2*E*), with the unfolding of the N-terminal transmembrane domain (TMD1), encompassing transmembrane (TM) helices one to six, alone contributing an extension increase of 57 nm (*SI Appendix*, Fig. S4*B*). Thus the 216 nm peak stabilized by lumacaftor aligned closely with the predicted extension of an intermediate where only TMD1 remains folded (*SI Appendix*, Fig. S4*B*). In addition, we computed sliding-window differences between histograms collected with and without the corrector (Fig. 1*G* and *SI Appendix*, Fig. S4 *C* and *D*; see *Methods* for the sliding window difference analysis). We used a 20-nm window—corresponding both to the typical spacing between unfolding histogram peaks and to the expected extension gained upon unzipping a TM-helix hairpin at 12 pN (*SI Appendix*, Fig. S4*D*). This analysis revealed a pronounced dwell-time increase centered at 217 nm (Fig. 1*G*), with significant positive deviations spanning 207 to 227 nm [Fig. 1*H* and *SI Appendix*, Fig. S4*E*; see *Materials and Methods* for the area under the curve (AUC) analysis]. Taken together, these data indicate that TMD1 unfolds last in our MT experiments.

Similarly, cross-correlation analysis indicated that the most prominent peak stabilized by ATP appeared around 157 nm in extension (precisely 158 nm or 156 nm depending on the second ligand) (Fig. 1*I* and *SI Appendix*, Fig. S4*F*). A comparative analysis of the unfolding profiles with and without ATP revealed a marked increase in dwell time (Fig. 1*J* and *SI Appendix*, Fig. S4*G*), with significant positive differences spanning the 157 to 177 nm region (Fig. 1*K*). Furthermore, the presence of ATP notably enhanced sampling in earlier extension regions: in particular, the extension region from 0 to 61 nm exhibited virtually no sampling in the absence of ATP (*SI Appendix*, Fig. S4*G*). Given that ATP binds to both NBD1 and NBD2 (Fig. 1*B* and *SI Appendix*, Fig. S5*A*), our observations suggest that the 157 to 177 nm region most likely corresponds to NBD1, which harbors the degenerate ATP-binding site defective of hydrolyzing ATP (41). We further hypothesize that the extension increase from 0 to 61 nm reflects unfolding of NBD2 and the lasso helices—an assignment that aligns with the pulling geometry of our MT assay, where unfolding is likely to initiate from either the N- or C-terminus.

To test this hypothesis, we first engineered a CFTR variant containing cysteine substitutions at Y109 and E1126 (Fig. 1*L* and *SI Appendix*, Fig. S1 *I*–*M*). This modification introduced a “knotted” topology that restricted unfolding to NBD2 and the lasso helices in the absence of the reducing agents (Fig. 1*L* and *SI Appendix*, Fig. S5*B*). The unfolding histogram of the “knotted” CFTR was compared to sliding windows of the WT CFTR unfolding histogram (in the presence of ATP) along the extension axis (Fig. 1*M*). This comparison revealed that the smallest difference between the histograms occurred when the WT CFTR signal was aligned to the 0 to 61 nm extension range (Fig. 1 *M* and *N* and *SI Appendix*, Fig. S5*C*). In a second test, we disrupted ATP binding to NBD2 by introducing the Y1219A mutation, which eliminates stacking interactions at the ATP site (*SI Appendix*, Fig. S5*A*). While ATP still enhanced the 156 nm peak (*SI Appendix*, Fig. S5 *D* and *E*), the Y1219A mutation significantly reduced dwell times in the 0 to 61 nm region (*SI Appendix*, Fig. S5 *F*–*H*). Together, these results support our hypothesis that the initial 61 nm extension arises from unfolding of NBD2 and the lasso helices (*SI Appendix*, Fig. S5*I*), while the extension increase from 157 to 206 nm predominantly reflects NBD1 unfolding (*SI Appendix*, Fig. S5*J*).

Finally, we identified a consistent valley in the unfolding histograms, spanning from 120 to 140 nm across all reaction conditions tested (*SI Appendix*, Fig. S5*K*). The absence of significant peaks in this region suggests the unfolding of a disordered segment, permitting us to infer that this region corresponds to the extension of CFTR’s R domain.

Together, these data allowed us to construct a detailed unfolding pathway for CFTR (Fig. 1*O*). Under a mechanical force of 12 pN, CFTR unfolds in a domain-specific sequence. The process begins with the unfolding of the N-terminal lasso helices and the C-terminal NBD2, followed by the six TM helices in TMD2. This is then succeeded by the unfolding of the R domain, NBD1, and finally TMD1.

### The CFTR Refolding Pathway.

**Fig. 2.**
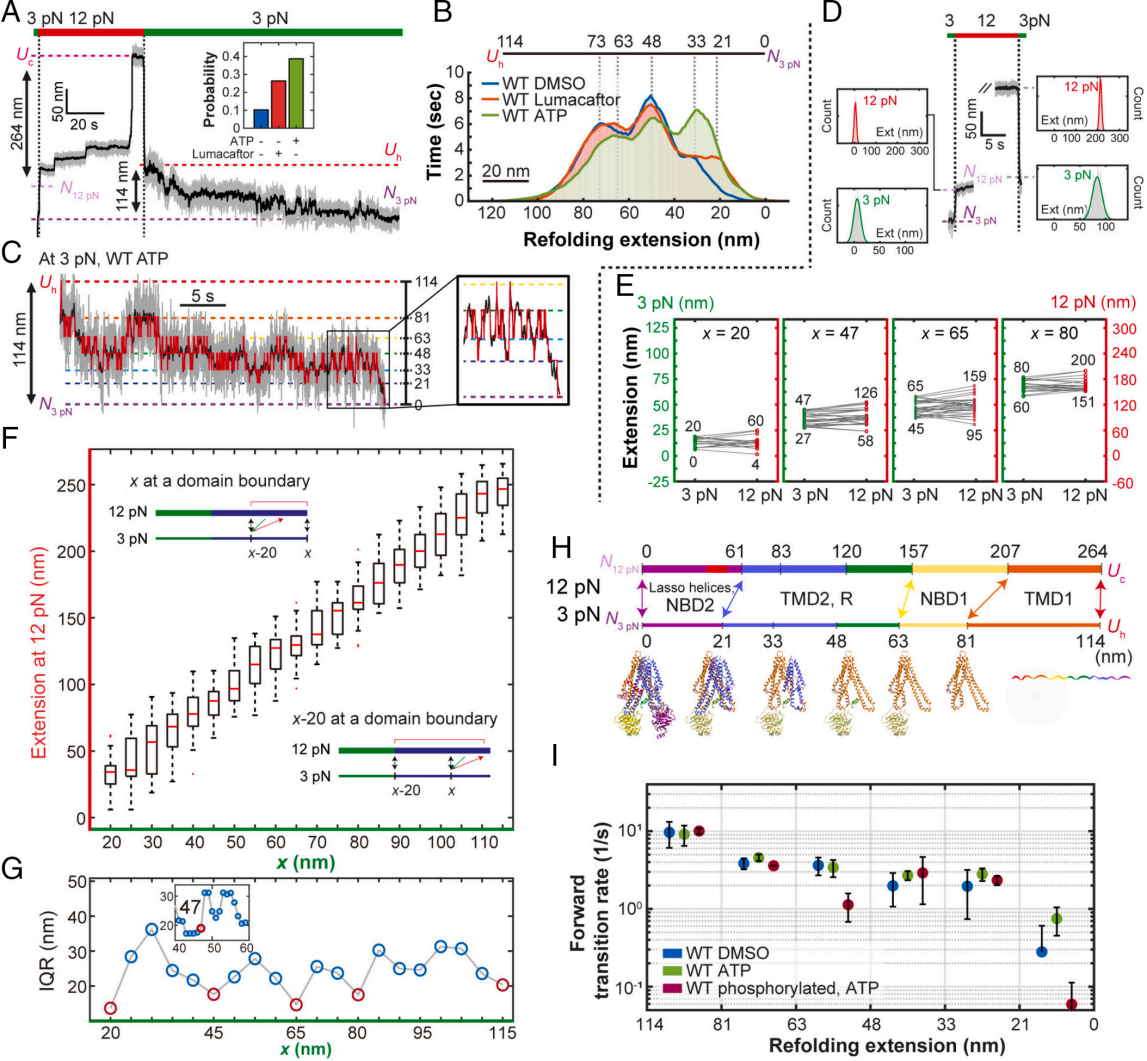
Single-molecule MT assay for CFTR refolding. (*A*) Experimental protocol for studying CFTR refolding dynamics. Inset shows the probability of complete refolding. (*B*) Refolding histograms generated with a 10-Hz median filter reveal multiple distinct peaks, corresponding to refolding intermediates. *n* = 27 (WT DMSO), *n* = 34 (WT ATP), and *n* = 32 (WT Lumacaftor). (*C*) Representative refolding trace with 1.2 kHz raw data (gray) and 10-Hz median filtered data (black). Viterbi path was overlaid on the data (red). Horizontal lines indicate extension values shown on the right. (*D*) Force-jump experiments to record extension states at 3 pN before and after a jump to 12 pN. (*E*) Examples of mapping corresponding extension values between 3 pN and 12 pN. Numbers below and above the green and red circles indicate the minimum and maximum extensions observed at 3 pN and 12 pN, respectively, for a given *x*. (*F*) Correlative mapping between 3-pN and 12-pN extension spaces. *n* = 164 force-jump traces. The *Inset* shows reduced variance occurs when *x* corresponds to a domain boundary (see *SI Appendix*, Fig. S6 *F* and *G* for detailed explanation). (*G*) interquartile range (IQR) for different values of *x*. Local minima correspond to distinct structural regions at 20, 65, 80, and 115 nm. Around *x* = 45 nm, detailed IQR analysis at 1-nm resolution uncovers a sharp increase in IQR above 47 nm (*Inset*). (*H*) Correlative comparison of unfolding intermediates at 12 pN and refolding intermediates at 3 pN. (*I*) Forward transition rates among refolding intermediates for nonphosphorylated CFTR in the presence of DMSO or ATP and for phosphorylated CFTR in the presence of ATP. Data represent means and SD for *n* individual traces. *n* = 27 for WT DMSO, *n* = 34 for WT ATP and *n* = 24 for WT phosphorylated, ATP.

We next studied the in vitro CFTR refolding pathway at a single-molecule level. Based on the measured 114 nm extension during refolding (Fig. 2*A* and *SI Appendix*, Fig. S6*A*) and our theoretical calculation (*SI Appendix*, Fig. S2*F*), we propose that upon force relaxation to 3 pN, CFTR rapidly transitions into an unfolded helical (*U*_h_) state, where only the helical secondary structures are restored (36, 37). The formation of tertiary structures follows over the course of minutes, with only a 10% probability of achieving the fully folded native (*N*_3pN_) state for the WT CFTR without ligands (Fig. 2 *A*, *Inset*). The addition of lumacaftor or ATP substantially increased the refolding probability to 26% and 39%, respectively (Fig. 2 *A*, *Inset*).

Refolding histograms, generated by compiling all the extension values sampled during these 3 pN refolding trials (with 10-Hz median filtering applied) displayed several peaks, indicative of distinct refolding intermediates (Fig. 2*B* and *SI Appendix*, Fig. S6*B*). To independently determine the positions of refolding intermediates, we analyzed individual refolding traces with a hidden Markov model (HMM) (Fig. 2*C* and *SI Appendix*, Fig. S6 *A* and *C*) (42, 43). Using the Bayesian Information Criterion (BIC), we identified five most likely intermediates, with extension values of 21, 33, 48, 63, and 81 nm, respectively (*SI Appendix*, Fig. S6 *C*, *Inset*). These values closely corresponded to the peaks observed in the refolding histograms (Fig. 2*B* and *SI Appendix*, Fig. S6*B*).

To explore the relationship between the 3 pN refolding intermediate states and the unfolding intermediates observed at 12 pN, we performed a force-jump experiment, rapidly alternating the applied force between 3 and 12 pN (Fig. 2*D*). We recorded the extension states immediately before and after each force jump, regardless of the jump direction (Fig. 2*D* and *SI Appendix*, Fig. S6*D*). The extension values at the two force levels showed a linear relationship, validating the force-jump method (*SI Appendix*, Fig. S6*E*). We then grouped the 3-pN extension states into 20-nm windows, with the upper limit of each window defined as *x* (e.g., *x* = 20 nm for a window spanning 0 to 20 nm) (Fig. 2*E*). Consistent with the linear correlation, the 20-nm windows at 3 pN corresponded closely to distinct clusters at 12 pN (Fig. 2 *E* and *F*). An intriguing observation is that the broadness of the 12-pN extension clusters, as measured by the IQR, varied periodically (Fig. 2*G*). The local minima, at *x* values of 20, 47, 65, and 80 nm, align closely with the refolding intermediates identified at 21, 48, 63, and 81 nm (Fig. 2*G* vs. Fig. 2 *B* and *C*). This alignment led us to hypothesize that the minimal spread at 12 pN occurred when *x* corresponds to a domain boundary (Fig. 2 *F*, *Insets*), as fully folded domains are more stable thereby exhibiting minimal structural changes during force jumps (*SI Appendix*, Fig. S6 *F* and *G*).

Furthermore, the mapping between 3 and 12 pN extensions along both directions of force-jump was asymmetric, extending into larger 12 pN values, rather than extending into the smaller 12 pN values (*SI Appendix*, Fig. S6 *H* and *I*). This observation supports the notion that the upper boundary of each 20-nm window at *x*, rather than the lower at *x*-20 nm, should align with domain boundaries, resulting in a tighter IQR (Fig. 2 *F*, *Insets* and *SI Appendix*, Fig. S6 *F* and *G*). Finally, analyzing the upper limits of the 12-pN extension clusters revealed positions at 60, 126, 159, 200, and 264 nm at 12 pN (*SI Appendix*, Fig. S6*J*, upper value of 12-pN extension). These values closely matched domain boundaries determined in high-force unfolding trials (Figs. 1*O* and 2*H*), suggesting that folding occurs in the reverse order of unfolding.

For structural identification of the refolding intermediate at 48 nm, which is a prominent peak in the refolding histogram, we compared the refolding kinetics of dephosphorylated CFTR vs. phosphorylated CFTR (Fig. 2*I*). The transition rates between intermediates, determined from the dwell times at each intermediate, progressively decreases as the protein refolds, with the 21-to-0 nm transition—representing NBD2 and lasso helices folding—being the slowest step (Fig. 2*I*). The early stages of folding—specifically transition from 114 to 81 nm—occurred rapidly (Fig. 2*I*), which accounts for the lack of a discernible 81 nm peak in the refolding histograms (Fig. 2*B* and *SI Appendix*, Fig. S6*B*). Phosphorylation of the R domain selectively altered the later stages of refolding, affecting transitions between 63 and 48 nm, and between 21 and 0 nm (Fig. 2*I*). Analyzing multiple possible scenarios (*SI Appendix*, Fig. S7 *A* and *B*) indicate that the 48 nm intermediate most likely represents an intermediate with the R domain assembled onto TMD1/NBD1 (*SI Appendix*, Fig. S7*B*, scenario 2-2). Supporting this hypothesis, cryo-EM studies indicated that the dephosphorylated R domain, although largely lacking defined secondary structures, packs along the inner surface of NBD1 (29).

Last, the observation that TMD2 completed its folded structure at the 21 nm extension led us to characterize the conformational state at 33 nm, where TMD2 appeared to be partially assembled, extending up to half of TM helix 10 (Fig. 2*H* and *SI Appendix*, Fig. S7*B*, scenario 2-2). It is particularly notable that the transition from 33 to 21 nm encompasses the completion of TMD2, including the formation of the TM helices 11 and 12 and its association with NBD1.

Collectively, these results provide an in vitro refolding pathway of CFTR, which closely mirrors that of the unfolding process (Fig. 2*H*). TMD1 folds first and rapidly, followed sequentially by NBD1, the R domain, TMD2, and finally, NBD2 along with the N-terminal lasso helices. The overall process has a low success rate, which is improved by the folding corrector lumacaftor and ATP, likely through the stabilization of TMD1 and the NBDs, respectively (Fig. 2 *A*, *Inset*).

### Unfolding and Refolding Pathways of ΔF508 CFTR.

In the cellular environment, the ΔF508 variant exhibits severe folding and trafficking defects that underlie the pathogenesis of cystic fibrosis (44–46). Cryo-EM structure of ΔF508 CFTR reveals that ΔF508 NBD1 is too flexible to stably associate with the TM helices (33). This defect further disrupts proper positioning of the R domain, as it packs onto the inner surface of NBD1 (33). We next asked whether these structural defects alter the unfolding and refolding pathways, creating alternative routes through a distorted folding energy landscape.

**Fig. 3.**
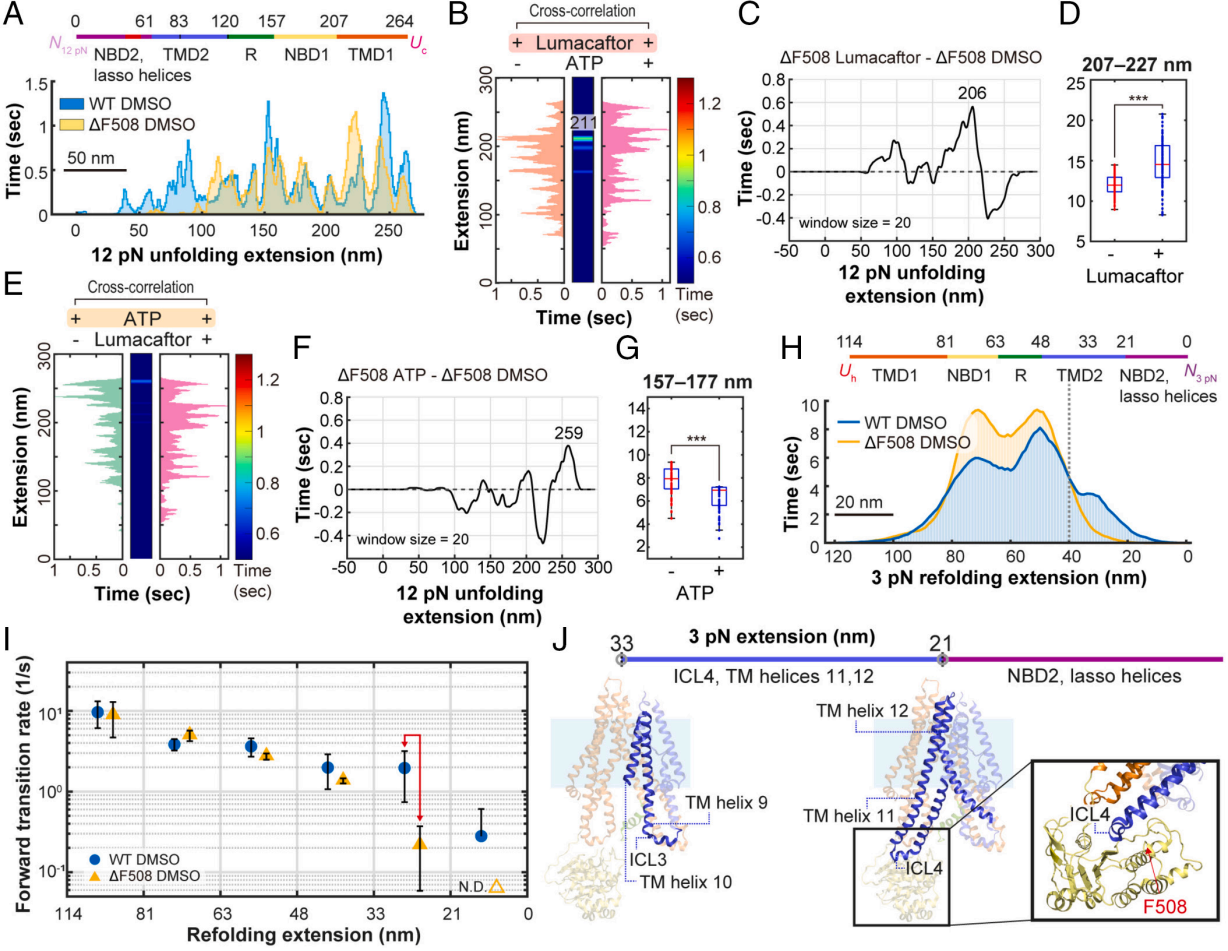
Single-molecule unfolding and refolding of ∆F508 CFTR. (*A*) Unfolding histograms for WT and ΔF508 CFTR in the absence of ligands but with DMSO. *n* = 48 (ΔF508 DMSO). (*B*) Cross-correlation analysis in the presence of lumacaftor, with or without ATP. *n* = 70 (ΔF508 Lumacaftor) and *n* = 50 (ΔF508 ATP + lumacaftor). (*C*) Sliding window difference analysis of ΔF508 CFTR in the presence of lumacaftor vs. DMSO. *n* = 70 (ΔF508 Lumacaftor) and *n* = 48 (ΔF508 DMSO). (*D*) Comparison of AUC in the 207 to 227 nm extension range with or without lumacaftor. (*E*) Cross-correlation analysis in the presence of ATP, with or without lumacaftor. *n* = 45 (ΔF508 ATP) and *n* = 50 (ΔF508 ATP + Lumacaftor). (*F*) Sliding window difference analysis in the presence of ATP vs. DMSO. *n* = 45 (ΔF508 ATP) and *n* = 48 (ΔF508 DMSO). (*G*) Comparison of AUC in the 157 to 177 nm extension range with versus without ATP. (*H*) Refolding histograms for WT and ΔF508 CFTR in the absence of ligands. *n* = 27 (WT DMSO) and *n* = 27 (ΔF508 DMSO). (*I*) Forward rate constants for folding transitions of WT and ΔF508 CFTR with DMSO. The red arrow highlights the effect of the ΔF508 mutation on the transition from 33 to 21 nm. *n* = 27 for ΔF508 DMSO. N.D., not determined. (*J*) Structural model of CFTR folding intermediates corresponding to extension values of 33 nm and 21 nm at 3 pN.

To measure the altered folding properties of the ΔF508 variant, we expressed and purified the dual-SpyTagged ΔF508 CFTR (*SI Appendix*, Fig. S8 *A*–*E*). Using the same force-ramp cycles, we observed that ΔF508 CFTR began unfolding at much lower force levels compared to WT CFTR, indicating reduced stability (compare Fig. 1*D* with *SI Appendix*, Fig. S9*A*). Despite this, ΔF508 CFTR displayed distinct unfolding intermediates very similar to those of the WT CFTR (Fig. 3*A*). Moreover, during unfolding under a constant 12 pN force, the addition of lumacaftor consistently resulted in a prominent cross-correlation peak at 211 nm (Fig. 3*B* and *SI Appendix*, Fig. S9*B*). Comparative analysis of the unfolding histograms with and without lumacaftor revealed that the dwell time in the extension region spanning 207 to 227 nm was prominently increased by lumacaftor (Fig. 3 *C* and *D* and *SI Appendix*, Fig. S9*C*). Thus, similar to WT CFTR, the final 57 nm extension observed during the unfolding of ΔF508 CFTR is likely attributable to the unfolding of TMD1 (*SI Appendix*, Fig. S4*B*). However, unlike WT CFTR, ATP did not enhance the unfolding peaks corresponding to NBD1 (i.e., between 157 and 206 nm extension), but instead caused a slight decrease (Fig. 3 *E*–*G* and *SI Appendix*, Fig. S9 *D* and *E*). These findings, consistent with prior studies (33, 47), indicate that the ΔF508 NBD1 was too flexible to be stabilized by ATP.

When the force was reduced to 3 pN, ΔF508 CFTR, like WT CFTR, began to refold in a stepwise manner (*SI Appendix*, Fig. S9*F*). While compiled refolding histograms and HMM analyses of individual refolding traces identified intermediates closely resembling those of WT CFTR, refolding predominantly stalled before reaching the 21 nm extension, highlighting the intrinsic defect of ΔF508 in achieving the fully folded state (Fig. 3*H* and *SI Appendix*, Fig. S9*F*). Notably, the unfolding histograms had revealed lack of peaks within the initial 100 nm extension region for ΔF508 CFTR (Fig. 3*A*). As refolding occurs in reverse order of unfolding, this structural region corresponds to the 0-to-40 nm range in the refolding histogram (Figs. 2*H* and 3 *A* and *H*). Supporting this interpretation, ΔF508 CFTR showed significantly reduced sampling below 40 nm compared to WT, underscoring the compromised stability of later-folding domains (Fig. 3*H*).

To dissect the folding defect of ΔF508 in details, we observed the occasional sampling of ΔF508 below 40 nm over extended periods and extracted transition rate constants to quantify which refolding steps were most affected by the ΔF508 (Fig. 3*I*). Contrary to expectations, the rate constant associated with NBD1 folding (81-to-63 nm transition), where F508 resides, remained largely unaffected (Fig. 3*I*). However, the forward transition from 33-to-21 nm, corresponding to the refolding of TM helices 11 and 12 in TMD2, was tenfold slower in ΔF508 CFTR (Fig. 3*I*, red arrow). Additionally, the backward rate, reflecting local unfolding, increased by twofold (*SI Appendix*, Fig. S10, *Right* plots). The rate constant for the final transition, involving the refolding and assembly of NBD2 and lasso helices, could not be determined for ΔF508 due to the limited sampling beyond the 21 nm extension (Fig. 3*I* and *SI Appendix*, Fig. S10, *Right* plots).

These observations were further substantiated by force-jump experiments (*SI Appendix*, Fig. S9 *G*–*I*). Analysis of the mapping broadness revealed local minima at 65, 80, and 105 nm (*SI Appendix*, Fig. S9 *I*, *Bottom*), which corresponded to 166, 198, and 260 nm in the 12-pN unfolding extension space (*SI Appendix*, Fig. S9*H*). These positions closely align with folding transitions associated with R domain, NBD1, and TMD1 respectively (Fig. 2*H*), which suggests that these domains were able to complete tertiary structure formation, albeit with reduced stability. In contrast, folding transitions associated with TMD2 and NBD2 were significantly diminished or absent (Fig. 3 *A*, *H*, and *I*), indicating a defect in forming stable tertiary structures in the C-terminal half of the protein. Together, these results indicate that ΔF508 CFTR largely follows the same folding trajectory as WT CFTR but exhibits selective destabilization of intermediates beyond NBD1, particularly in the TMD2/NBD2 region.

Given that ΔF508 CFTR largely follows the same folding trajectory as WT CFTR, the impaired 33-to-21 nm transition suggests that the absence of F508 severely impairs the refolding of TM helix 11 and downstream structural elements (Figs. 2 *H* and 3*J*). In the WT protein, the aromatic side chain of F508 interacts with hydrophobic residues in TM helix 11 (33). Our data suggest that these interactions are crucial for the proper folding of TM helix 11, and when TM helix 11 fails to fold correctly, the folding of downstream structural elements is also compromised (Fig. 3*J*) (17, 33, 48, 49). These observations strongly support a template-based folding pathway and suggest that the primary folding defect in ΔF508 lies in the inability of NBD1 to properly guide the folding of TM helix 11.

### Small-Molecule Correctors Rescue the ΔF508 CFTR Folding Defect At 33-to-21 nm Transition.

**Fig. 4.**
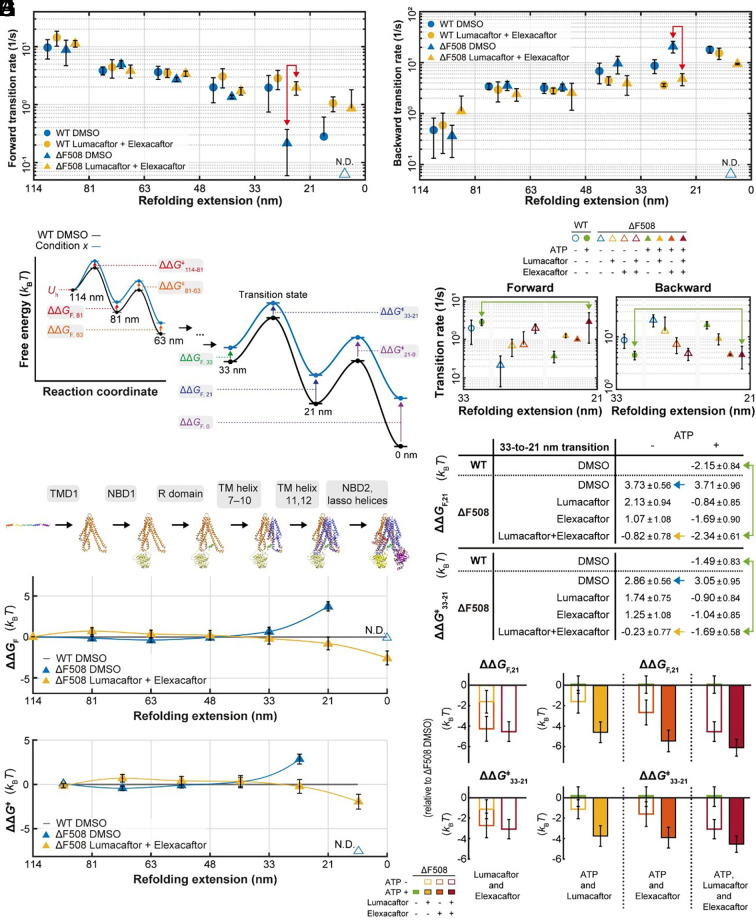
Folding impairment of ΔF508 CFTR and its correction by the folding correctors. (*A* and *B*) Forward (*A*) and backward (*B*) transition rates for WT and ΔF508 CFTR, with and without lumacaftor and elexacaftor. *n* = 29 for WT Lumacaftor + Elexacaftor and *n* = 39 for ΔF508 Lumacaftor + Elexacaftor. N.D., not determined. (*C*) Schematic representation of ΔΔ*G*^‡^ and ΔΔ*G*_F_. The black curve represents the free energy landscape of WT CFTR in DMSO, and the blue curve represents the free energy landscape in the condition being examined. (*D* and *E*) ΔΔ*G*_F_ (*D*) and ΔΔ*G*^‡^ (*E*) calculated relative to WT DMSO (gray line at 0), comparing ΔF508 DMSO and ΔF508 with Lumacaftor + Elexacaftor. Data represent means and propagated errors calculated from transition rate values obtained from *n* individual traces (*n* values in *SI Appendix*, Fig. S10). N.D., not determined. (*F*) Forward and backward transition rates for the 33-to-21 nm transition for WT and ΔF508 CFTR, with various combinations of lumacaftor, elexacaftor, and ATP. (*G*) ΔΔ*G*_F,21_ and ΔΔ*G*^‡^_33-21_ values for WT and ΔF508 CFTR, calculated relative to WT DMSO. Data represent means and propagated errors for *n* individual traces, with exact *n* values provided in *SI Appendix*, Fig. S10. (*H* and *I*) Comparative analysis of ΔΔ*G* values at 33-to-21 nm transition to evaluate the combinatorial effects of lumacaftor and elexacaftor (*H*) and of the combinatorial effects of ATP and the folding correctors (*I*). ΔΔ*G* values are calculated relative to ΔF508 DMSO.

Having identified the impaired folding of TM helix 11 as the key defect in ΔF508 CFTR, we tested whether folding correctors could restore this transition. While ΔF508 in DMSO stalled beyond 40 nm during refolding, the addition of correctors enabled progression through the final transitions (*SI Appendix*, Fig. S11*A*), ultimately achieving full refolding in a subset of molecules, more closely resembling WT behavior under the same treatment conditions (*SI Appendix*, Fig. S11*B*). HMM analysis revealed that the intermediate states in corrector-treated ΔF508 overlapped substantially with those of WT, although residual instability at the 33-to-21 nm transition persisted under certain conditions (*SI Appendix*, Fig. S11 *C*–*F*). Reflecting this, the overall refolding success probability remained lower than that of WT; nonetheless, the addition of ATP and the correctors significantly increased the probability of reaching the fully folded state (*SI Appendix*, Fig. S11*G*). Notably, kinetic analysis revealed that lumacaftor and elexacaftor specifically accelerated the 33-to-21 nm transition, corresponding to the folding of TM helices 11 and 12, and restored the folding disrupted in ΔF508 (Fig. 4*A*, red arrow). These correctors also reduced the frequency of backward transitions, indicating a stabilization of folding intermediates (Fig. 4*B*, red arrow). Together, these findings confirm that the primary folding defect in ΔF508 lies in the inefficient refolding of TM helix 11 and its downstream elements, and demonstrate that folding correctors act by enhancing the folding rate at this critical step while preserving the folding pathway.

We next undertook a quantitative analysis to delineate how the ΔF508 mutation and its pharmacological correction reshape the CFTR folding energy landscape. To this end, we determined 228 rate constants—encompassing both forward and backward transitions—across various conditions (*SI Appendix*, Fig. S10). These kinetic parameters enabled precise estimation of changes in free energy barriers (ΔΔ*G*^‡^) and relative shifts in the free energies of refolding intermediates (ΔΔ*G*_F_) (*SI Appendix*, Fig. S12). Conceptually, increased forward rates reflect catalytic enhancement of folding transitions, while decreased backward rates indicate stabilization of folded conformations (*SI Appendix*, Fig. S13*A*). Therefore, we attributed variations in forward rates primarily to changes in ΔΔ*G*^‡^, and those in backward rates to a combination of altered ΔΔ*G*^‡^ and ΔΔ*G*_F_ (relative to WT CFTR in DMSO, unless otherwise specified) (Fig. 4*C*). Notably, because the free energy states of intermediate conformations are inherently linked across folding steps, ΔΔ*G*_F_ values derived from an earlier transition worked as initial conditions for calculating ΔΔ*G*^‡^ and ΔΔ*G*_F_ in the subsequent step (Fig. 4*C* and *Materials and Methods* for free energy analysis).

**Fig. 5.**
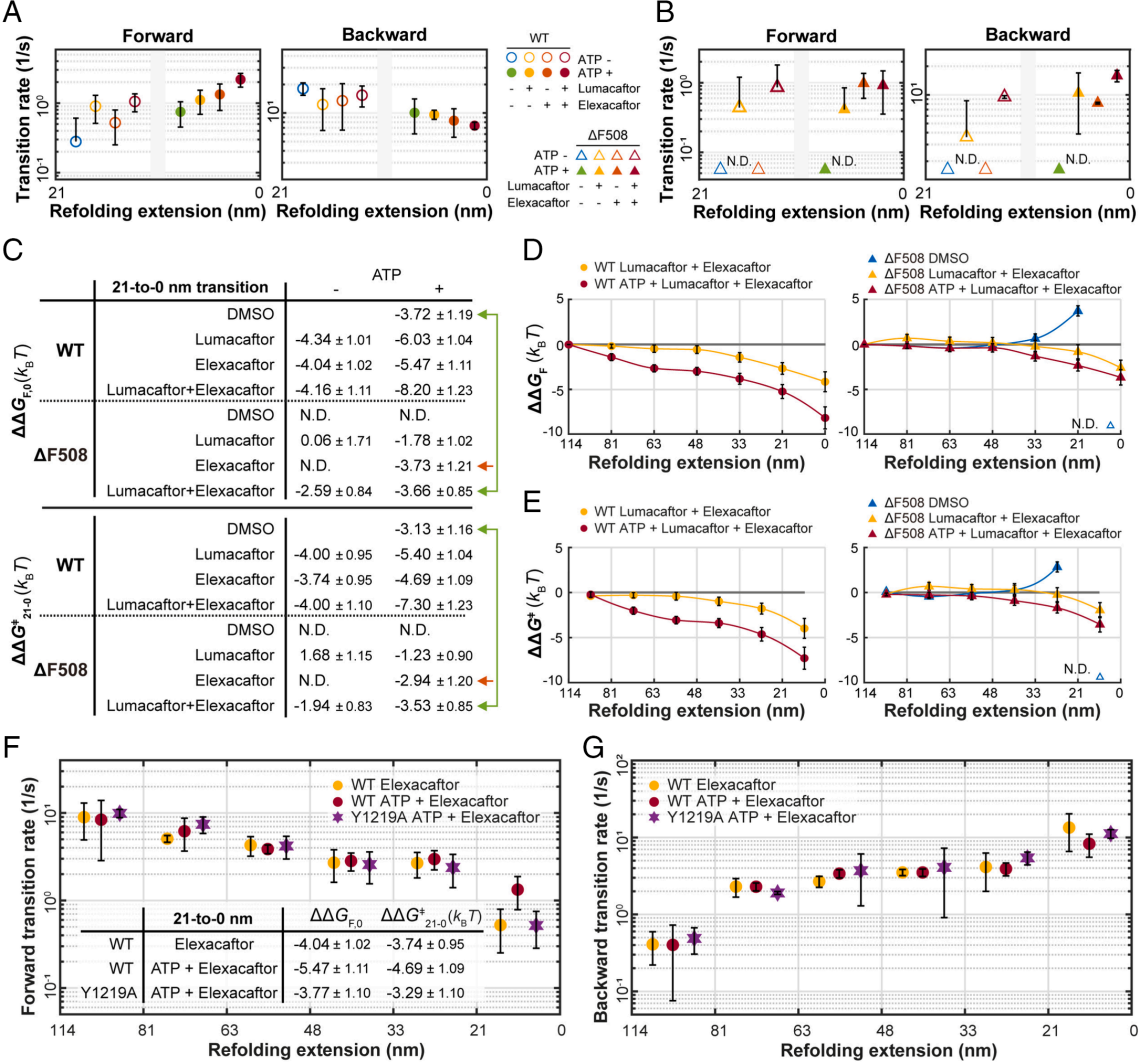
Small-molecule correctors rescue the ΔF508 CFTR folding defect at 33-to-21 nm transition. (*A* and *B*) Forward and backward transition rates for the 21-to-0 nm transition for WT (*A*) and ΔF508 (B) with various combinations of lumacaftor, elexacaftor, and ATP. (*C*) ΔΔ*G*_F,0_ and ΔΔ*G*^‡^_21-0_ values for WT and ΔF508 CFTR, calculated relative to WT DMSO. (*D* and *E*) ΔΔ*G*_F_ (*D*) and ΔΔ*G*^‡^ (*E*) calculated relative to WT DMSO (gray line at 0), comparing WT Lumacaftor + Elexacaftor and WT ATP + Lumacaftor + Elexacaftor (*Left*), ΔF508 Lumacaftor + Elexacaftor and ΔF508 ATP + Lumacaftor + Elexacaftor (*Right*). (*F* and *G*) Forward (*F*) and backward (*G*) transition rates for refolding transitions of Y1219A and WT CFTR with the indicated conditions. *n* = 45 for Y1219A ATP + Elexacaftor. ΔΔ*G*_F,0_ and ΔΔ*G*^‡^_21-0_ values for Y1219A, compared with WT CFTR (*F*, inset). The exact *n* values are provided in *SI Appendix*, Fig. S10.

Using this approach, we systematically mapped how ΔΔ*G*_F_ and ΔΔ*G*^‡^ accumulated along the folding pathway for both WT and ΔF508 CFTR, and how these energy profiles were modulated by folding correctors and ATP (Fig. 4 *D* and *E* and *SI Appendix*, Fig. S12). The ΔF508 mutation severely destabilized the 21-nm refolding intermediate (ΔΔ*G*_F,21_), increasing its free energy by 3.73 *k*_B_*T* (Fig. 4 *D*, *F* and *G*, blue symbols and an arrow). This destabilization propagated through the folding pathway in an amplified manner, ultimately rendering the free energy of the fully folded state (ΔΔ*G*_F,0_) undeterminable under the mutant condition (Fig. 4*D*). In addition, the ΔF508 mutation impaired the 33-to-21 nm transition itself, elevating the corresponding free energy barrier (ΔΔ*G*^‡^_33-21_) by 2.86 *k*_B_*T* (Fig. 4 *E*–*G*, blue symbols and an arrow). While either lumacaftor or elexacaftor alone partially mitigated these defects, their combined application effectively restored ΔΔ*G*_F,21_ and ΔΔ*G*^‡^_33-21_ to −0.82 *k*_B_*T* and −0.23 *k*_B_*T*, respectively, values closely aligning with those of WT CFTR in DMSO (Fig. 5 *D*–*G*, yellow symbols and arrows; WT reference shown in *SI Appendix*, Fig. S13 *B* and *C*).

Notably, in reducing ΔΔ*G*_F,21_ and ΔΔ*G*^‡^_33-21_, lumacaftor and elexacaftor exerted additive effects, with the effects being especially pronounced in ΔF508 CFTR (Fig. 4*H*; WT data shown in *SI Appendix*, Fig. S13*D*). This mechanistic synergy likely underlies the basis for the most widely used CFTR modulator therapy, which combines lumacaftor (or tezacaftor) and elexacaftor with the potentiator ivacaftor (50). Furthermore, lumacaftor, elexacaftor, and their combination all exhibited strong synergy with ATP in promoting the 33-to-21 nm transition (Fig. 4*I*; WT data shown in *SI Appendix*, Fig. S13*E*). Structural studies suggest that lumacaftor binds to TMD1 (34), whereas elexacaftor binds the lasso helices and TM helices 10 and 11 (33). Thus, our data suggest that lumacaftor, elexacaftor, and ATP bind distinct structural elements in ΔF508 CFTR to effect additive rather than redundant rescue of folding and interdomain assembly. The combined application of all three restored the forward and backward 33-to-21 nm transition rates of ΔF508 CFTR to values comparable to those determined for WT CFTR in the presence of ATP alone (Fig. 4 *F* and *G*, green arrows), achieving a near-complete rescue of this critical transition.

### Rescue of the Terminal Folding Step in WT and ΔF508 CFTR.

We next examined the final 21-to-0 nm transition, which corresponds to the folding of NBD2 and the lasso helices. Lumacaftor, elexacaftor, ATP, and their combinations exhibited pronounced restorative effects on this transition in both WT and ΔF508 CFTR (Fig. 5 *A*–*C*). These findings are consistent with our earlier observation that this step represents the slowest and most recalcitrant transition, even in WT CFTR (Fig. 2*I*), and therefore possesses the greatest potential for energetic improvement (Fig. 5 *D* and *E* and *SI Appendix*, Fig. S12).

As the destabilizing effect of the ΔF508 mutation propagated through the folding pathway, the stabilizing influence of folding correctors on refolding intermediates also accumulated progressively in WT CFTR (Fig. 5 *D* and *E* and *SI Appendix*, Fig. S12*A*). In the absence of ATP, the effects of the correctors became evident beyond the 48 nm extension (Fig. 5 *D* and *E* and *SI Appendix*, Fig. S12*A*, yellow circles), corresponding to TMD2 and subsequent domains. ATP further amplified these stabilizing effects, culminating in the largest ΔΔ*G* reduction at the 21-to-0 nm transition and highlighting the synergistic interplay between correctors and ATP (Fig. 5 *D* and *E* and *SI Appendix*, Fig. S12*A*, red circles). Notably, in ΔF508 CFTR, the cumulative energetic gains from folding correctors and ATP were quantitatively smaller than those observed in WT CFTR (Fig. 5 *D* and *E*, compare *Left* and *Right* panels). This underscores the importance of the structural integrity of preformed domains in effectively propagating the effects of correctors through the folding pathway. Collectively, these observations support a template-driven model in which the effects of folding correctors propagate through downstream folding transitions, rather than acting solely at isolated structural elements.

This model was further supported by detailed energetic analysis of the 21-to-0 nm transition. The large negative values of ΔΔ*G*_F,__0_ and ΔΔ*G*^‡^_21-0_ determined for WT CFTR indicated that correctors and ATP actively catalyzed this final transition, rather than merely accelerating preceding steps (Fig. 5*C*). Although baseline ΔΔ*G* values could not be measured for ΔF508 CFTR in the presence of DMSO due to the paucity of complete refolding events, the energetic effects of pharmacological correctors could still be inferred. Notably, while elexacaftor alone had minimal effect on the 21-to-0 nm transition, its combination with ATP reduced ΔΔ*G*_F,__0_ by −3.73 *k*_B_*T* and ΔΔ*G*^‡^_21-0_ by −2.94 *k*_B_*T* (Fig. 5*C*, orange arrows). Importantly, this synergy was abolished by the Y1219A substitution in WT CFTR, which disrupts ATP binding to NBD2 (Fig. 5 *F* and *G*), indicating that elexacaftor’s effect depends on direct ATP interaction with NBD2 rather than an allosteric influence from NBD1. Although elexacaftor binds distally from NBD2, these results underscore the critical role of interdomain communication in CFTR folding and reveal that effective pharmacological rescue requires not only local stabilization but also long-range energetic coupling between structural domains.

Finally, in the presence of both folding correctors and ATP, the ΔΔ*G*_F,__0_ and ΔΔ*G*^‡^_21-0_ values for ΔF508 CFTR reached −3.66 and −3.53 *k*_B_*T*, respectively, bringing these values close to those observed for WT CFTR with ATP (Fig. 5*C*, green arrows). While the synergistic effects of lumacaftor and elexacaftor, in the presence of ATP, appeared attenuated during the folding of NBD2 and the lasso helices (Fig. 5*C*, compare orange and green arrow; WT data shown in *SI Appendix*, Fig. S13 *F* and *G*)—possibly reflecting a convergence of their mechanisms at this terminal stage—the final and rate-limiting folding transition was fully restored to WT-like levels through the combined action of folding correctors and ATP.

## Discussion

In this study, we examined the unfolding and refolding processes of individual CFTR molecules in lipid bicelles by applying mechanical force to completely unfold the fully folded protein, and then relaxing the force to observe its refolding. This process differs substantially from in vivo CFTR biosynthesis, where folding occurs through both co-translational and posttranslational phases and involves a network of molecular chaperones and protein quality control mechanisms (51–54). Nevertheless, we show that native-like CFTR structures can be achieved in in vitro refolding experiments and the probability of successful refolding is sensitive to ATP and folding correctors. These data provide direct support for Anfinsen’s thermodynamic hypothesis that protein structure is determined solely by its amino acid sequence (55). This finding is particularly significant given CFTR’s large, multidomain architecture.

By quantitatively analyzing thermodynamic and kinetic aspects of the CFTR refolding pathway, we propose that CFTR exhibits a hierarchical, templated folding pathway where the N-terminal TMD1 and NBD1 fold first and act as scaffolds for the subsequent folding of their C-terminal counterparts, TMD2 and NBD2. Although ΔF508 CFTR follows the same pathway as WT CFTR, the lack of F508 at the NBD1/TMD2 interface reduces the stability of NBD1, but more importantly, it slows the folding of TMD2 by an order of magnitude. Furthermore, weakened NBD1/TMD2 interface in ΔF508 CFTR destabilizes both TMD2 and NBD2, even after folding is complete, as evidenced by the total disappearance of early unfolding peaks within the first 100 nm extension range at 12 pN. These defects ultimately obstruct the formation of the native conformation and weaken structural connectivity across CFTR.

Although our proposed template-driven model deviates from the traditional view that individual CFTR domains fold separately before assembling into the native-like conformation (53, 54), it aligns well with many observations reported in the literature. During CFTR biosynthesis, individual domains—except for NBD2—rapidly adopt conformations that render them resistant to proteolysis (16, 48, 53, 56). By contrast, NBD2 folding is not only slow but also dependent on the presence of other domains (54, 57–59). Furthermore, in the early co-translational stage, NBD1 already interacts with TMD1 (60, 61) and the formation of the most stable TMD2 conformation requires both TMD1 and NBD1 (54). These findings support that in the cellular environment, CFTR folding also follows a template-driven pathway rather than an independent domain-folding model.

Crucially, this templated folding mechanism implies that the free energy states of individual folding intermediates are intimately coupled along the CFTR folding trajectory. Although all kinetic measurements were necessarily performed under finite mechanical load, these transition rates were measured at the lowest force compatible with reliable resolution, which likely brackets the mechanical regime experienced by nascent membrane proteins during translation and chaperone-assisted folding. The resulting folding energy landscape—determined by the 228 forward and backward transition rates spanning distinct folding steps under various pharmacological and ATP conditions—revealed that the impact of the ΔF508 mutation was not confined to NBD1 but instead propagated throughout the entire folding pathway. Notably, the destabilizing effect became increasingly amplified beyond 33 nm, affecting transitions associated with TMD2, NBD2, and the lasso helices. In parallel, the effects of folding correctors were also transduced along the folding trajectory and were further potentiated by ATP. The combined action of lumacaftor and elexacaftor—particularly when administered with ATP—robustly enhanced the final folding transitions in ΔF508 CFTR, ultimately restoring their energetics to levels comparable to WT.

Our data further reveal that folding correctors act not only by stabilizing folded domains but also by actively catalyzing specific folding transitions, as evidenced by reductions in the energy barriers. This dual mode of action suggests that folding correctors and ATP serve as allosteric modulators that reshape the folding energy landscape—functioning in a chaperone-like manner to facilitate otherwise inefficient transitions, rather than merely locking preexisting folded conformations. Furthermore, the quantitative synergy analysis suggests that lumacaftor, elexacaftor, and ATP engage distinct structural elements and likely reinforce separate interfaces critical for the assembly of its C-terminal counterparts. Interestingly, the synergistic action between lumacaftor and elexacaftor was diminished during the final folding step involving NBD2 and the lasso helices, possibly reflecting a convergence of their actions at this terminal stage. Together, these findings support a multifaceted mechanism, wherein distinct yet complementary modulators can act in concert with ATP to restore native folding—highlighting a therapeutic strategy rooted in energetic complementation.

The experimental platform described here offers a powerful framework for interrogating how CFTR mutations disrupt the folding process, pinpointing the folding transitions that a given set of mutations imposes its most severe energetic penalty. As demonstrated, the system is amenable to testing novel corrector candidates and quantifying their ability to resuscitate specific folding transitions, even in rare CFTR mutations, particularly those unresponsive to current correctors and potentiators. These insights can inform the mechanistic understanding of how specific mutations perturb folding energetics and how correctors restore function—thereby laying the groundwork for extending CFTR-targeted therapies and advancing treatments for other protein folding disorders.

## Materials and Methods

### Expression and Purification of the Human CFTR.

Dual-SpyTagged CFTR fused to a C-terminal PreScission Protease (PPX)-cleavable GFP tag was cloned into the BacMam vector and expressed as previously described for the WT CFTR (6). For purification of the Y109C/E1126C CFTR variant, DTT was omitted from all buffers. For purification of the ΔF508 CFTR variant, 1 mM ATP was added to extraction and wash buffers. A dialysis step was added prior to gel filtration to remove lumacaftor and elexacaftor copurified with CFTR from the culture medium. The eluted sample was dialyzed for a total of 48 h against wash buffer with several buffer exchanges.

### Patch-Clamp Electrophysiology.

Recordings were performed using the inside-out patch configuration with local perfusion at the patch. Dual-SpyTagged CFTR was activated by 300 nM PKA phosphorylation with 3 mM ATP. Borosilicate glass pipettes were pulled from capillaries (outer diameter 1.5 mm, inner diameter 0.86 mm, Sutter) to 1.5 to 2.5 MΩ resistance and fire polished. Membrane potential was clamped at −30 mV. Macroscopic currents were recorded at 25 °C using an Axopatch 200B amplifier, a Digidata 1550 digitizer and the pClamp software suite. Recordings were low-pass-filtered at 1 kHz and digitized at 20 kHz. The displayed recordings were further low-pass filtered at 100 Hz. Data were analyzed with Clampfit.

### Preparation of DNA Handles.

The preparation of SpyCatcher-conjugated DNA followed an established protocol (37, 39). In summary, 512-base-pair DNA fragments carrying biotin or digoxigenin modification at one end and an amine group at the other were amplified by PCR. These DNA fragments were reacted with SM(PEG)_2_ using an amine-sulfhydryl crosslinking reaction for 1 h at room temperature. Following DNA maxiprep purification, the fragments were conjugated to purified SpyCatcher/Maltose Binding Protein through a thiol-maleimide crosslinking reaction, which proceeded overnight at 4 °C. To eliminate unconjugated SpyCatcher and unconjugated DNA, the resultants were purified using anion exchange chromatography with a 1 mL Mono Q column (GE Healthcare) followed by amylose affinity chromatography (New England BioLabs).

### Single-Molecule MT Experiments.

WT or mutant CFTR proteins were mixed with the SpyCatcher-DNA handles in 1.3% (w/v) bicelle buffer and incubated for 90 min at room temperature. For the experiments, 0.02% (w/v) streptavidin-coated polystyrene particles were first injected into a homemade flow cell consisting of two cover slips. Next, 15 nM Neutravidin was injected and incubated for 8 min. Then the chamber was washed with bicelle buffer prior to sample injection. The bicelle buffer was supplemented with ligands according to experimental conditions (e.g., ATP diluted in bicelle buffer for ATP conditions or lumacaftor diluted in bicelle buffer for lumacaftor conditions). The final ligand concentrations were ATP, 1 mM; MgCl_2_, 2 mM; lumacaftor, 1 µM; elexacaftor, 1 µM. CFTR conjugated with DNA handles was then introduced into the chamber and incubated for 20 min, followed by buffer exchange to remove unbound protein. Finally, magnetic beads were injected and incubated for 1 h.

### Extensible Worm-Like Chain (eWLC) Model.

The FECs for DNA and unstructured polypeptides were analyzed using the eWLC model, which characterizes the mechanical response of semiflexible biopolymers under applied tension. The eWLC equation is given by

$$F=\left(\frac{k_{B}T}{l_{p,i}}\right)\left[\frac{1}{4{\left(1-z_{i}/\left(n_{i}l_{c,i}\right)\right)}^{2}}-\frac{1}{4}+\frac{z_{i}}{n_{i}l_{c,i}}-\frac{F}{K_{i}}+\sum_{j=1}^{j\leq 7}a_{j}{\left(\frac{z_{i}}{n_{i}l_{c,i}}-\frac{F}{K_{i}}\right)}^{j}\right],$$

where the index i denotes either DNA or unstructured polypeptide (p). Here, $k_{B}T$ represents the thermal energy, $l_{c}$ and $l_{p}$ correspond to the contour length and persistence length, respectively ($l_{c,\mathrm{DNA}}=0.338\;\mathrm{nm}$, $l_{c,p}=0.36\;\mathrm{nm}$ and $l_{p,\mathrm{DNA}}=38.5\;\mathrm{nm}$, $l_{p,p}=0.39\;\mathrm{nm}$). The elastic modulus values are $K_{p}∼50$ µN and $K_{DNA}∼500$ pN. F is the applied force and $a_{j}$ are polynomial coefficients for improved approximation. The total number of monomers in each component is defined as follows: $n_{DNA}=512$ for each handle, $n_{linker,p}=54$ for polypeptide linker between CFTR and the DNA handle, and $n_{\mathrm{CFTR},p}=1,426$ for the CFTR protein.

### Kessler-Rabin (KR) Model.

To describe the helical states, *U*_h_, the KR model was used,

$$z_{h}≃-\frac{1}{2f}-\frac{\chi }{f\tanh2\chi }+\frac{L_{h}}{\tanh2\chi }-\frac{2\chi^{2}}{3f}\left(\frac{1}{\tanh fL_{h}}-\frac{fL_{h}}{(\sinh fL_{h}{)}^{2}}-1\right),$$

where $\chi \equiv \sqrt{f\frac{{\left(n_{h}l_{c,h}\right)}^{2}}{4l_{p,h}}}$, $f\equiv \frac{F}{k_{B}T}$ and $n_{h}$ is the number of amino acids consisting of the transmembrane helix. The persistence length $\left(l_{p,h}\right)$ is 9.17 nm and the contour length ${(l}_{c,h})$ along helical axis is averagely 0.16 nm per amino acid.

### Extension Value Calculations.

The extensions expected for the *U*_c_ and *U*_h_ states are described as follows,

$$z_{m}=z^{\mathrm{handle}}+z^{\mathrm{CFTR}}={\left(z_{p}+z_{\mathrm{DNA}}\right)}^{\mathrm{handle}}+z_{p\;\mathrm{or}\;h}^{\mathrm{CFTR}},$$

where $z_{m}$ is the measured extension, $z_{p}$ represents the extension of the unstructured polypeptide linker between DNA handle and the target protein (linkers from each end of the protein to SpyCatcher), $z_{DNA}$ corresponds to the extension of the DNA in the DNA handle, and $z_{porh}^{CFTR}$ is the total molecular extension of CFTR with contributions from unstructured and/or helical parts. The $z_{p}$ and $z_{DNA}$ values are inversely calculated from the eWLC model at given force levels, and $z_{porh}^{CFTR}$ corresponds to the total molecular extension of CFTR. The values of $z_{p}^{CFTR}$ and $z_{h}^{CFTR}$ are derived from the eWLC and KR model, respectively.

To understand how the extension values correspond to which intermediate states, we calculated the number of amino acids that undergo stretching and the change in the end-to-end distance of the remaining structured regions. The increase in extension for an intermediate state $z_{i}^{CFTR}$ is proportional to the number of unfolded amino acids (${\Delta n}_{i}$). This value is obtained by multiplying the number of unfolded amino acids by the extension per amino acid at a given force. In summary, the relationship used for calculating the measured extension value at an intermediate state *i* ($z_{m,i}$) is

$$z_{m,i}=\left(z_{i}^{\mathrm{CFTR}}-d_{\mathrm{before}}+d_{\mathrm{after}}\right),$$

where *d*_before_ and *d*_after_ represent the end-to-end distance of the structured regions before and after the transition, respectively.

### Cross-Correlation Analysis.

To assess the common effect of the ligands under different conditions (i.e., Lumacaftor effect in WT Lumacaftor and WT ATP + Lumacaftor), we applied a cross-correlation method by computing the product of corresponding peak values at each extension. A matrix was generated, where each element was calculated as

$$C\left(i\right)=\frac{\sum_{k=\max(1,i-5)}^{\min(264,i+5)}\sqrt{H_{A,i}\times H_{B,k}}+\sum_{k=\max(1,i-5)}^{\min(264,i+5)}\sqrt{H_{A,k}\times H_{B,i}}-\sqrt{H_{A,i}\times H_{B,i}}}{\mathrm{count}}.$$

$C\left(i\right)$ represents the correlation product at position *i* (0 < *i* < 264) and $H_{X,i}$ corresponds to the histogram height at position *i* for condition of *X* (*A* or *B*). The count is the number of elements included in the sum for normalization purposes. We included values within *i* ± 5 nm to account for peak widths.

### Sliding Window Difference Analysis.

To identify regions exhibiting ligand-induced changes in dwell time, we performed pairwise histogram subtraction between conditions differing by a single ligand (e.g., to assess the effect of Lumacaftor, the WT Lumacaftor histogram was subtracted from the WT DMSO histogram). Since TM segments generally unfold as helical hairpins, and the typical extension of a hairpin at 12 pN is approximately 20 nm, we applied a 20-nm sliding window to the histograms to detect such localized effects.

### AUC Comparison Analysis.

For each condition, we randomly selected 40 unfolding cycles to generate a histogram, which was sufficient to observe divergence in the resulting histograms. This sampling process was repeated 100 times to calculate AUCs over defined extension ranges corresponding to distinct structural domains: 30 to 50 nm (NBD2 and lasso helices), 157 to 177 nm (NBD1), and 207 to 227 nm (TMD1). The boxplot elements represent the following: median (red vertical line); box limits (25th and 75th percentiles); whiskers (extending to 1.5× IQR). Statistical significance was tested using a two-sample *t* test (***P ≤ 1 × 10^−3^).

### HMM Analysis.

The adjustable parameters in our system are the number of states (*n*), the extension position for *i*-th intermediate state ($\overset{-}{Zi}$), and the transition matrix of rates between states ($\overset{↔}{k}$). The optimal number of states (*n*) was obtained from BIC: BIC = *q*ln(*N*)—2ln($\hat{L}$) where *q* is the number of output parameters given by model, *N* is sample size and $\hat{L}$‚ is the maximum value of the likelihood function. Maximum likelihood estimation was performed using the Baum–Welch algorithm. The extension traces were median-filtered with 10-Hz window, and the extension position/deviation for each state was estimated from the Gaussian Mixture Model in the HMM analysis. Finally, the resulting traces were verified by the Viterbi algorithm.

### Force Jump Analysis.

Force-jump experiments were performed by rapidly increasing and decreasing the force between 3 pN and 12 pN while monitoring the magnetic bead’s position in the extension space. Extensions were recorded at each force, and histograms were generated with Gaussian fitting. The peak of each histogram was used as the representative extension value. A 20-nm moving window was applied across the 3-pN extension space. This window size was chosen based on the minimum extension observed for an individual domain, which was approximately 20 nm. A box plot was constructed using the 25th to 75th percentile range, and IQR values were analyzed to assess variability.

### Free Energy Analysis.

Relative free energy differences (ΔΔ*G*^‡^ and ΔΔ*G*_F_) were calculated with respect to WT CFTR under DMSO conditions. Free energy differences for each transition from the *n*th state, *i_n_*nm to the (*n*+1)th state, *i*_n+1_ nm were determined iteratively using

$$\begin{array}{c} \Delta \Delta G_{x,\;\left(i_{n}-i_{n+1}\right)}^{‡}=\Delta G_{x,\;\left(i_{n}-i_{n+1}\right)}^{‡}-\Delta G_{WT\;DMSO,\;\left(i_{n}-i_{n+1}\right)}^{‡}+\Delta \Delta G_{F_{x,i_{n}}}\\ =-k_{B}T\ln\frac{k_{f_{x,\left(i_{n}-i_{n+1}\right)}}}{k_{f_{WT\;DMSO,\;\left(i_{n}-i_{n+1}\right)}}}+\Delta \Delta G_{F_{x,i_{n}}}, \end{array}$$

$$\begin{array}{c} \Delta \Delta G_{F_{x,i_{n+1}}}=\Delta G_{F_{x,i_{n+1}}}-\Delta G_{F_{WT\;DMSO,i_{n+1}}}+\Delta \Delta G_{F_{x,i_{n}}}\\ =-k_{B}T\ln\frac{k_{f_{x,\;\left(i_{n}-i_{n+1}\right)}}}{k_{f_{WT\;DMSO,\;\left(i_{n}-i_{n+1}\right)}}}+k_{B}T\ln\frac{k_{b_{x,\;\left(i_{n}-i_{n+1}\right)}}}{k_{b_{WTDMSO},\;\left(i_{n}-i_{n+1}\right)}}+\Delta \Delta G_{F_{x,i_{n}}}, \end{array}$$

where *n* ranges from one to six, with the corresponding intermediate states: *i*_1_ = 114 nm, *i*_2_ = 81 nm, *i*_3_ = 63 nm, *i*_4_ = 48 nm, *i*_5_ = 33 nm, and *i*_6_ = 21 nm. *x* represents the condition being examined. For *n* = 1, ${\Delta \Delta G}_{F_{x,i^{n}}}=0$, as the energy of the *U*_h_ state (114 nm) is equivalent across all the conditions, including WT and ∆F508 CFTR. *k*_f_ and *k*_b_ represent the forward and backward transition rates respectively. Both ΔΔ*G*^‡^ and ΔΔ*G*_F_ are expressed in units of *k*_B_*T.*

## Supplementary Material

Appendix 01 (PDF)

## Data Availability

Code used for data analysis have been deposited in Github (https://github.com/tyyoonlab-snu/Magnetic-Tweezers-2024) (62). All data that support the findings of this study are available in the manuscript and/or *SI Appendix*.
